# Less invasive surfactant administration and complications of preterm birth

**DOI:** 10.1038/s41598-018-26437-x

**Published:** 2018-05-29

**Authors:** Christoph Härtel, Pia Paul, Kathrin Hanke, Alexander Humberg, Angela Kribs, Katrin Mehler, Matthias Vochem, Christian Wieg, Claudia Roll, Egbert Herting, Wolfgang Göpel

**Affiliations:** 1 Department of Pediatrics, University of Lübeck, Lübeck, Germany; 2 Department of Neonatology, University of Cologne, Cologne, Germany; 3 Department of Neonatology, Olga Hospital Stuttgart, Stuttgart, Germany; 4 Department of Neonatology, Klinikum Aschaffenburg, Aschaffenburg, Germany; 5 Department of Neonatology, Vest Children’s Hospital Datteln, University Witten-Herdecke, Witten-Herdecke, Germany

## Abstract

In a large cohort study of the German Neonatal Network (GNN) we aimed to evaluate whether less invasive surfactant administration (LISA) strategy is associated with complications of preterm birth. Within the observational period n = 7533 very-low-birth-weight infants (VLBWI) with gestational age 22 0/7 to 28 6/7 weeks were enrolled in GNN; n = 1214 VLBWI never received surfactant, n = 2624 VLBWI were treated according to LISA procedure, n = 3695 VLBWI had surfactant via endotracheal tube (ETT). LISA was associated with a reduced risk for adverse outcome measures including mortality [odds ratio (OR) 0.66 (95% CI: 0.51–0.84), p < 0.001] bronchopulmonary dysplasia [BPD; OR 0.55 (95% CI: 0.49–0.62), p < 0.001], intracerebral hemorrhage (ICH) grade II-IV [OR 0.55 (95% CI: 0.48–0.64), p < 0.001] and retinopathy of prematurity [ROP; OR 0.62 (95% CI: 0.45–0.85), p < 0.001]. Notably, LISA was associated with an increased risk for focal intestinal perforation [FIP; OR 1.49 (95% CI: 1.14–1.95), p = 0.002]. The differences in FIP rates were primarily observed in VLBWI born <26 weeks (LISA: 10.0 vs. ETT: 7.4%, p = 0.029). Our observational data confirm that LISA is associated with improved outcome. In infants <26 weeks we noted an increased risk for FIP. Future randomized controlled trials including LISA need to integrate safety analyses for this particular subgroup.

## Introduction

Less invasive surfactant administration (LISA) to spontaneously breathing preterm infants has been reported to reduce mechanical ventilation, BPD and severe complications of prematurity in randomised controlled trials^1–3^, observational studies^4–6^ and recent meta-analyses^7–10^ as compared to intubation for surfactant delivery. Applying LISA to extremely preterm infants followed by non-invasive respiratory support, is a paradigm shift, the proposed effects of which need to be examined carefully. Several factors seem important to achieve a clinical benefit through LISA strategy: infant’s tolerance of surfactant administration while spontaneously breathing, efficacy of continuous positive airway pressure (CPAP)/non-invasive positive pressure ventilation (NIPPV), adequate breathing efforts - supported by methylxanthine treatment to prevent apnea of prematurity - and tolerance of enteral nutrition. Therefore, the LISA strategy represents a bundle of less invasive respiratory care procedures which has been adopted as primary surfactant mode of administration in many German neonatal units. To address further safety aspects of LISA strategy we aimed to evaluate in a large cohort study of the German Neonatal Network (GNN) whether LISA strategy is associated with less frequent complications of preterm birth in most susceptible infants born <29 weeks of gestation.

## Results

### Primary respiratory care

In our study cohort n = 1214 infants never received surfactant, n = 2624 VLBWI were treated with surfactant according to LISA method, n = 3695 VLBWI had surfactant treatment via endotracheal tube (ETT). As outlined in Table 1, the three groups differed significantly with regard to clinical characteristics. Specifically, infants who received surfactant by LISA or ETT were younger at birth, more often small-for-gestational age and suffered more often from moderate or severe RDS (peak FiO_2_ in the first 12 h) than infants who received no surfactant. The LISA procedure has been increasingly used in GNN centers during the observational period, e.g. 2009 vs. 2016: 28.7% vs. 50.1% of surfactant treated infants had LISA (Suppl. Figure 1).

**Table 1:** Clinical characteristics of the study population according to respiratory management/surfactant administration.

Clinical characteristics	No surfactant	LISA	Surfactant ETT	p	all
Number of infants	1214	2624	3695		7533
Gestational age (weeks), mean (SD)	27.4 (1.4)	26.8 (1.5)	26.2 (1.6)	<0.001*	26.6 (1.6)
Birth weight (g), mean (SD)	984 (234)	885 (290)	814 (244)	<0.001*	866 (266)
**Mode of birth (%)**				<0.001	
Spontaneous delivery	13.8	8.7	11.5		10.9
Caesarean section, elective	76.1	81.0	72.8		76.2
Caesarean section, emergency	10.0	10.3	15.6		12.9
**Cause of preterm birth (%)**					
Preterm labour	47.1	40.1	42.6	0.049	42.5
Amniotic infection syndrome	33.7	29.2	28.8	0.7	29.8
Pathological Doppler/Growth restriction	11.9	17.5	15.7	0.05	15.7
Pathological CTG	18.0	17.9	18.9	0.3	18.4
Pre-eclampsia	4.3	7.1	5.5	0.008	5.8
HELLP syndrome	4.6	8.4	7.4	0.12	7.3
Placental abruption	7.7	7.4	9.9	<0.001	8.7
**Apgar scores 5 min/10 min, mean (SD)**	8(1)/9(1)	8(1)/9(1)	7(2)/8(1)	<0.001*	7(2)/8(1)
Umbilical artery pH, mean (SD)	7.33 (0.09)	7.32 (0.09)	7.31 (0.11)	<0.001*	7.32 (0.1)
SGA (< 10th percentile, %)	6.4	11.6	15.5	<0.001*	12.6
Female gender (%)	50.5	46.0	45.2	0.5	46.3
Multiple birth (%)	28.7	33.4	30.9	0.04	31.4
Inborn (%)	96.8	98.4	95.2	<0.001	96.6
Antenatal steroids administered (%)	93.8	93.4	87.9	<0.001	90.8
German maternal background (%)	68.8	72.3	73.8	0.2	72.5
**Peak FiO2 in first 12h (%)**				<0.001	
21–39%	81.9	51.7	40.3		51.0
40–59%	14.0	29.1	27.3		25.8
60–100%	4.1	19.2	32.4		23.2
**Drug treatment of PDA (%)**	20.1	47.0	46.7	0.8	42.5
Indomethacin	7.6	18.4	19.3	0.4	17.1
Ibuprofen	14.5	28.9	33.3	<0.001	28.7
**Postnatal steroids (%)**	6.8	17.9	33.6	<0.001	23.9
Hydrocortisone	4.9	13.4	25.7		18.1
Dexamethasone	1.4	3.9	11.7		7.3
Prednisolone	2.0	4.0	5.6		4.5

p-values (LISA vs. Surfactant ETT) are derived from Pearson-chi^2^ test or Mann-Whitney-U-test if indicated (*).

### LISA is superior to intubation for surfactant delivery for short-term outcomes

In univariate analyses, LISA was superior to intubation for several clinical outcomes including clinical and culture-confirmed sepsis, pneumonia, higher grade ICH, PVL,ROP, Patent Ductus arteriosus (PDA) surgery, BPD and death but not for FIP and necrotizing enterocolitis (NEC). The proposed benefits of LISA were confirmed in multivariable logistic regression models for clinical sepsis, pneumonia, mortality, BPD, ICH grade II-IV, PVL, PDA and ROP (Table 2).

**Table 2:** Outcomes according to respiratory management/surfactant administration.

Clinical characteristics	No surfactant	LISA	Surfactant ETT	p*	Adjusted OR* (95% CI); p	Adjusted OR^1–12^ (95% CI); p	all
Number of infants	1214	2624	3695				7533
Clinical sepsis	27.4	34.9	46.3	<0.001	0.76 (0.68–0.85); p<0.001	0.86 (0.74–0.99); p = 0.048^1^	39.3
Blood-culture proven sepsis	11.6	14.6	19.6	<0.001	0.87 (0.75–1.0) p = 0.053	1.0 (0.83–1.21); p = 0.9^2^	16.6
Pneumonia	2.0	4.7	8.0	<0.001	0.67 (0.54–0.84) p = 0.001	0.68 (0.51–0.81); p = 0.012^3^	5.8
Intracerebral hemorrhage grade II-IV	4.8	12.9	24.3	<0.001	0.55 (0.48–0.64); p<0.001	0.62 (0.53–0.73); p<0.001^4^	17.2
Periventricular leukomalacia	2.4	3.6	5.5	<0.001	0.72 (0.56–0.94); p = 0.015	0.75 (0.56–1.01); p = 0.06^5^	4.3
PDA, surgical ligation	2.9	4.0	9.8	<0.001	0.51 (0.41–0.65); p<0.001	0.59 (0.44–0.74); p<0.001^6^	6.7
ROP requiring therapy (%)	2.4	4.3	8.5	<0.001	0.62 (0.45–0.85); p = 0.003	0.67 (0.48–0.94); p = 0.002^7^	6.1
FIP requiring surgery (%)	1.2	4.3	4.0	0.5	1.49 (1.14–1.95); p = 0.003	1.42 (1.06–1.89), p = 0.018^8^	3.7
NEC requiring surgery (%)	2.1	3.6	4.4	0.13	1.09 (0.83–1.43); p = 0.5	1.26 (0.94–1.68); p = 0.13^9^	3.7
BPD (%)	12.2	21.6	37.6	<0.001	0.55 (0.49–0.62); p<0.001	0.62 (0.54–0.72); p<0.001^10^	27.9
BPD or death (%)	13.7	24.5	43.9	<0.001	0.5 (0.44–0.57); p<0.001	0.58 (0.5–0.67); p<0.001^11^	32.3
Death (%)	2.1	4.1	7.8	<0.001	0.66 (0.51–0.84); p<0.001	0.76 (0.58–0.99); p = 0.039^12^	5.6

p-values (LISA vs. Surfactant ETT) are derived from Pearson-chi^2^ test;

*Adjusted OR indicate the effect of surfactant therapy LISA versus endotracheal tube (ETT) and were derived from multivariable logistic regression models including gestational age (per week), small-for-gestational age (SGA), gender, multiple birth, inborn, antenatal steroids, surfactant LISA or ETT.

^1–12^Adjusted ORs indicate the effect of surfactant therapy LISA versus ETT derived from regression models including known risk factors for the respective short term outcomes; specifically:

^1, 2, 3^gestational age (per week), SGA, gender, multiple birth, inborn, inotropes first 24 hours, amniotic infection syndrome, anhydramnios >5 days before birth, antenatal steroids, surfactant LISA or ETT.

^4^gestational age (per week), small-for-gestational age, gender, multiple birth, inborn, inotropes first 24 hours, amniotic infection syndrome, antenatal steroids, indomethacin prophylaxis, mode of delivery (spontaneous, elective Caesarean section, emergency Caesarean section), sepsis, surfactant LISA or ETT.

^5^gestational age (per week), small-for-gestational age, gender, multiple birth, inborn, inotropes first 24 hours, amniotic infection syndrome, antenatal steroids, surgery NEC or FIP, sepsis, surfactant LISA or ETT.

^6,9^gestational age (per week), small-for-gestational age, gender, multiple birth, inborn, inotropes first 24 hours, amniotic infection syndrome, antenatal steroids, surfactant LISA or ETT.

^7, 10–12^gestational age (per week), small-for-gestational age, gender, multiple birth, inborn, inotropes first 24 hours, amniotic infection syndrome, antenatal steroids, surgery NEC or FIP, sepsis, surfactant LISA or ETT.

^8^gestational age (per week), small-for-gestational age, gender, multiple birth, inborn, inotropes first 24 hours, amniotic infection syndrome, antenatal steroids, postnatal steroids, PDA treatment with indomethacin or ibuprofen, surfactant LISA or ETT.

### LISA strategy is associated with focal intestinal perforation in extremely preterm infants

**Figure 1:**
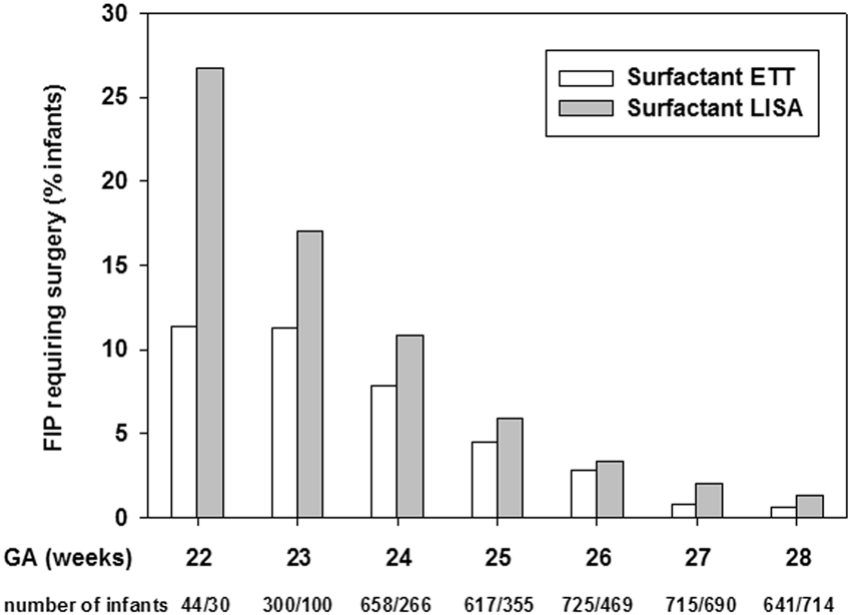
Incidence of FIP stratified to primary surfactant management. The figure depicts the percentage of infants suffering from FIP per week of gestation according to exposure to surfactant treatment: Surfactant via ETT (white bars), Surfactant via LISA (grey bars). Numbers of infants are given below each column, i.e. surfactant treated infants via ETT/surfactant treated infants via LISA in each week of gestation.

**Figure 2:**
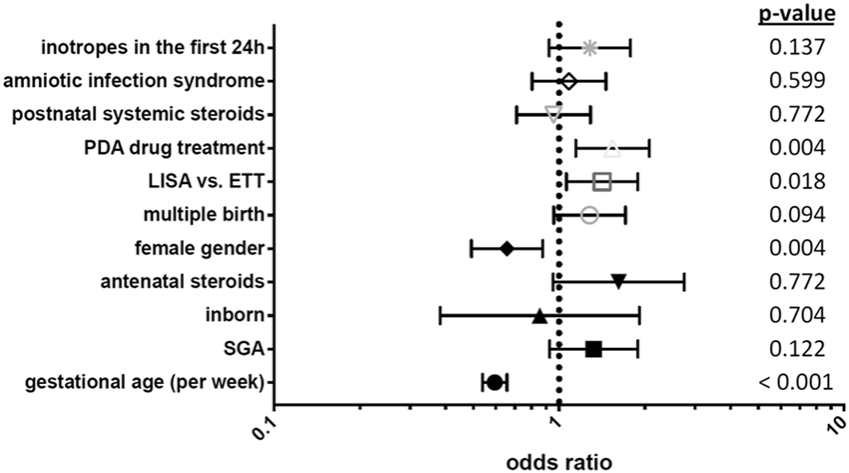
FIP requiring surgery – logistic regression model. The figure depicts the data of a multivariable logistic regression model to adjust the effect of LISA strategy for known or probable confounding variables including inotropes in the first 24 hours (surrogate marker for severity of primary compromise at birth), amniotic infection syndrome as cause of preterm birth, postnatal steroid hormones (dexamethasone, hydrocortisone, (methyl)prednisolone), PDA drug treatment (indomethacin, ibuprofen), multiple birth, female gender, antenatal steroids, inborn, SGA, gestational age per week. The symbols and lines describe the odds ratios and 95% confidence interval.

VLBWI without surfactant treatment had a low frequency of FIP (1.2%). VLBWI treated with LISA (4.3%) and VLBWI treated with surfactant via ETT (4.0%) had a comparable FIP rate. In the subgroup of infants with a gestational age <26 weeks, however, LISA treated infants had a higher risk for FIP as compared to infants receiving surfactant via ETT [75/751 (10.0%) vs. 119/1619 (7.4%), p = 0.029; Fig. 1)] but not in the subgroup of infants born 26–28 weeks [39/1873 (2.1%) vs. 30/2081 (1.4%), p = 0.13]. In a multivariable logistic regression analysis, surfactant administration with LISA was associated with an increased risk for FIP [odds ratio (OR) 1.49 (95% CI: 1.14–1.95), p = 0.003] as compared to surfactant treatment via ETT (reference). In a second regression model including further potential confounders such as clinical amniotic infection syndrome, inotropes in the first 24 hours, postnatal steroid exposure or drug PDA treatment we confirmed the independent association of LISA with FIP [OR 1.42 (1.06–1.89), p = 0.018; Fig. 2]. PDA drug treatment (indomethacin or ibuprofen) proved to be a risk factor for FIP [OR 1.53 (1.14–2.06), p = 0.005; Fig. 2]. When PDA treatment with single drugs was included in the regression models, the exposure to indomethacin [OR 1.66 (1.22–2.25), p = 0.001] but not ibuprofen [OR 1.03 (0.77–1.38), p = 0.9] was associated with FIP. However, the effect of LISA on FIP risk remained unchanged, i.e. regression models including indomethacin: OR 1.5 (1.12–2.02), p = 0.006; ibuprofen: OR 1.53 (1.14–2.04), p = 0.004].

## Discussion

LISA is a distinct feature of the approach to respiratory care to preterm infants in several countries^1–6^. The LISA procedure has been increasingly used in GNN centers during the observational period. With more than 2500 LISA treated infants this is the largest cohort report so far. We confirmed that LISA is superior to surfactant delivery via ETT with regard to several outcomes related to lung and CNS complications after preterm birth (1–10; 14). In addition, our data suggest beneficial effects regarding the risks of clinical sepsis, pneumonia and higher grade retinopathy. In the subgroup of extremely preterm infants <26 weeks, however, the LISA strategy was associated with a higher incidence of FIP requiring surgery.

FIP is a spontaneous single intestinal perforation typically found at the terminal ileum. Based on previous observational data gestational age is the predominating endogenous risk factor for FIP which occurs in 2–3% of VLBWI and in 5% of extremely-low-birth weight infants (ELBWI; birth weight <1000 g). The median gestational age of affected infants is 25–27 weeks. Male infants are at higher risk than female infants while and infants born SGA have a marked susceptibility to FIP due to predisposition for gut ischemia^11–13^. In addition, administration of postnatal steroids and non-steroidal anti-inflammatory drugs (specifically indomethacin in our setting) has been related to FIP development. Other frequently discussed but not confirmed risk factors for preterm infants are exposure to antenatal steroids, multiple birth and chorioamnionitis^12–14^. Epidemiological data indicate that FIP is associated with significant mortality and long-term morbidity^15–18^. In a recent prospective analysis of the Vermont-Oxford Network 19% of infants with laparotomy-confirmed FIP died, with a case-fatality rate of 26–31% in infants with a birth weight <750 g^19^. Although survival from FIP has increased over the past decades, the pathophysiology of FIP is still not very well understood^18^. The novel association between LISA and FIP observed in our large cohort needs to be discussed in the context of the complex management of highly susceptible infants. LISA itself may not be causative in the pathogenesis of FIP. A more likely association is that extremely preterm babies (especially those <26 weeks), who would previously have been intubated, are now being managed on CPAP (or NIPPV) at a much earlier and more vulnerable stage than ever before, facilitated by the dose of surfactant given via LISA soon after birth^20^. Several factors in line with this management need to be considered:

Firstly, maximum CPAP levels (±LISA) applied to highly vulnerable babies may have an impact. In a small retrospective cohort study of VLBWI placed on high noninvasive respiratory support (airway pressure ≥10 cm H_2_O for at least 12 continuous hours) using nasal continuous positive airway pressure (NCPAP) and/or nasal high-frequency ventilation (NIHFV, n = 70) no increase of FIP was noted^20^. In line with this, nasal continuous positive airway pressure may affect pre- and postprandial intestinal blood flow velocity in preterm infants^21^. Hence future prospective studies need to capture the exposure to relatively high positive pressure applied to the upper airway as an input variable.

Secondly, extremely vulnerable infants are exposed to several modulators of mucosal integrity including devices (ventilation support, gastric tubes), bacterial colonization, nutrition and drugs. The timing of treatment strategies - not only drugs (non-steroidal anti-inflammatory drugs, steroids) but also implementation of invasive measures (e.g. tracheal ventilation) - might be critical for FIP risk.

Thirdly, abdominal distension of the gastrointestinal tract due to delayed meconium passage and CPAP may result in increased shear forces and stretching of the intestinal mucosa. The texture of the gastric tube (size of side holes, closed or open tip) may be important for adequate aspiration of gas and stomach content in order to avoid massive dilatation of bowels. Finally, center specific aspects may also be significant, i.e. centers use different strategies in treating RDS and apnea/bradycardia syndrome with regard to the time point of secondary tracheal ventilation, particularly in infants <26 weeks. In line with this, the frequency of episodes with relevant desaturations might contribute to FIP risk via temporary hypoxia of the gut. Our data provide a basis for benchmarking and critically reviewing all aspects of less invasive surfactant application strategies. Strengths of our study are the large sample size and prospectively recorded datasets. With increasing expertise of NICUs in the technique and evidence that LISA is highly beneficial for several outcomes, we noted that an increasing number of infants <26 weeks are managed as such^22^. The major limitations of our study are the post-hoc analysis and the observational design. We are not able to rule out the possibility of unrecognized confounders [e.g. approach to neonatal resuscitation, changing strategies of PDA-management (ibuprofen, indomethacin, paracetamol), center aspects] which might bias the results of our analysis. Whether “protective” or earlier intubation of extremely preterm infants with significant abdominal distension is beneficial, needs to be subject of further trials. In line with this, animal models such as preterm lambs may complement clinical investigations and guide future research to evaluate underlying causes of our observation^23^.

In conclusion, our observational data confirm that LISA is associated with improved outcome. In highly preterm infants <26 weeks we noted an increased risk for FIP. Future randomized controlled trials including LISA strategy need to integrate safety analyses for this particular subgroup, and clinicians need to balance the optimal time point of secondary tracheal ventilation in extremely preterm babies initially managed with LISA.

## Methods

The GNN is a population-based cohort study of VLBWI enrolled in 54 neonatal intensive care units in Germany (GNN). The data for this observational investigation were collected between the 1^st^ of January 2009 until the 31^st^ of December 2016. After written informed consent was given by the parents, infants were enrolled in the GNN by the attending physicians. Then a predefined clinical data set was recorded on case report forms and sent to the GNN coordinating center in Lübeck. The inclusion criteria for this observational study were infants with a birth weight <1500 g and gestational age ≥22 0/7 and <29 weeks who received primary intensive care. We excluded VLBWI who were previously enrolled in RCTs evaluating LISA strategy, i.e. AMV and NINSAPP^1,2^, and infants with lethal malformations.

A physician or study nurse from the central GNN office (University of Lübeck) with expertise in neonatology monitored the data quality by annual site visits. Clinical data were coded and entered into a central database. Written parental consent was obtained by parents or caregivers. The GNN study was approved by the ethics committee at each participating centre.

### Ethics

All experimental protocols were approved by the ethics committee of the University of Lübeck(08–022) and the local ethical committees at each study center. Informed consent was obtained from all subjects. All methods were carried out in accordance with relevant guidelines and regulations, specifically: the Declaration of Helsinki, the current revision of ICH Topic E6, the Guidelines for Good Clinical Practice, and the Guidelines of the Council for International Organization of Medical Sciences, the WHO (“Proposed International Guidelines for biomedical research involving human subjects”).

## Definitions

### Outcomes

*Clinical sepsis* was defined as sepsis with at least two signs (temperature >38 °C or <36.5 °C, tachycardia >200/min, new onset or increased frequency of bradycardias or apneas, hyperglycemia >140 mg/dl, base excess <−10 mval/l, changed skin color, increased oxygen requirements) and one laboratory sign (C-reactive protein >1 mg/dl, immature/neutrophil ratio >0.2, white blood cell count <5/nl, platelet count <100/nl) and antibiotic treatment for ≥5days, but no proof of causative agent in the blood culture. *Blood-culture confirmed sepsis* was defined as clinical sepsis with proof of causative agent in the blood culture^24^.

*Pneumonia* was defined as infection with at least one radiological sign (e.g. infiltrate on X-ray) or new deterioration of gas exchange (frequent desaturations, increased oxygen requirements) and at least four clinical/laboratory signs, i.e. tachycardia >200/min, new onset or increased frequency of bradycardias or apneas, new onset of tachypnea/dyspnea, putrid tracheal aspirate, more frequent need of tracheal aspiration, temperature instability; C-reactive protein >2 mg/dl, immature/neutrophil ratio >0.2^24^.

*Moderate to severe intracerebral haemorrhage (ICH)* was defined as grade II–IV ICH according to Papile^25^.

*Periventricular leukomalacia (PVL)* was defined as white-matter brain injury, characterized by cystic degeneration of white matter near the lateral ventricles as diagnosed by ultrasound imaging which was applied in all participating centres.

*Retinopathy of prematurity (ROP)* was defined as higher stage ROP requiring intervention (crytherapy, laser therapy or anti-VEGF treatment).

*FIP requiring surgery* was defined as the occurrence of spontaneous intestinal perforation with the need for laparotomy and the macroscopic diagnosis of isolated FIPs as described by the attending surgeon.

*NEC requiring surgery* was defined as clinical NEC classified as Bell Stage II or Bell Stage III with the need for laparotomy with or without resection of necrotic gut, and the macroscopic diagnosis of NEC.

*Patent ductus arteriosus (PDA) surgery* was defined as required surgical ligation of PDA.

*Bronchopulmonary dysplasia* (BPD) was defined as need for oxygen supplementation or ventilation support at 36 weeks corrected age.

*Death* was defined as mortality during primary stay in hospital.

### Clinical Parameters

*Gestational age* was calculated from the best obstetric estimate based on early prenatal ultrasound and obstetric examination. Small for gestational age was defined as birth weight percentile <10 according to gestational age^26^.

*Severity of RDS* was characterized as categories of peak FiO_2_ in the first 12 h, i.e. mild RDS: FiO_2_ 0.21–0.39, moderate RDS: FiO_2_ 0.4–0.59, severe RDS: FiO_2_ ≥0.6.

*Drug treatment of Patent Ductus arteriosus* (PDA) was defined as treatment of PDA with ibuprofen or indomethacin.

*Postnatal steroid treatment* was defined as any systemic treatment with steroids (hydrocortisone, (methyl-) prednisolone or dexamethasone).

### Statistical analysis

Univariate analysis: Study populations were compared using univariate techniques. Continuous variables (gestational age, birth weight, Apgar scores) were evaluated with Mann-Whitney-U test. Categorical variables (e.g. gender) were evaluated with a two-tailed Pearson-Chi-square test.

Multivariate analysis: Logistic regression analyses were performed for all outcomes subjected to univariate analysis to adjust the effect of LISA for known confounding variables, particularly gestational age per week, small-for-gestational age, inborn, antenatal steroids, gender and multiple birth. Mode of surfactant administration was included as an independent categorical variable with surfactant treatment via ETT as reference. To address independent factors associated with FIP we additionally included reported or assumed risk factors for FIP requiring surgery based on literature^11–13,27^ such as amniotic infection syndrome as cause of preterm birth, inotropes in the first 24 hours, exposure to postnatal steroids and exposure to drug treatment of PDA. Odds ratios (OR) and 95% confidence intervals (CI) were calculated. A p-value of <0.05 was considered statistically significant. Missing data were not imputed. Data analysis was performed using the SPSS 22.0 data analysis package (Munich, Germany).

## Electronic supplementary material

supplemental information
