# Supplementary material for: Less invasive surfactant administration and complications of preterm birth
Source: Sci Rep. 2018 May 29;8:8333. doi: 10.1038/s41598-018-26437-x (PMC5974027; doi:10.1038/s41598-018-26437-x)
Supplement: Supplementary file 1 — supplemental information [file 41598_2018_26437_MOESM1_ESM.docx]

**Supplemental information – Table 1**

**Less invasive surfactant administration and complications of preterm birth**

Christoph Härtel^1^, MD, Pia Paul^1^, MD, Kathrin Hanke^1^, MD, Alexander Humberg^1^, MD, Angela Kribs^2^, MD, Katrin Mehler^2^, MD, Matthias Vochem^3^, MD, Christian Wieg^4^, MD, Claudia Roll^5^, MD, Egbert Herting^1^, MD, PhD, and Wolfgang Göpel^1^, MD

^1^Department of Pediatrics, University of Lübeck, Germany

^2^Department of Neonatology, University of Cologne, Germany

^3^Department of Neonatology, Olga Hospital Stuttgart, Germany

^4^Department of Neonatology, Klinikum Aschaffenburg, Germany

^5^Department of Neonatology, Vest Children’s Hospital Datteln, University Witten-Herdecke, Germany

Corresponding author:

Christoph Härtel

Department of Paediatrics, University of Lübeck,

Ratzeburger Allee 160, 23538 Lübeck, Germany

Tel.: + 49-451-500 2685, FAX: + 49-451-500 6222

E-mail: [christoph.haertel@uksh.de](mailto:haertel@paedia.ukl.mu-luebeck.de)

**Running title:** LISA is associated with FIP in preterm infants

Supplemental figure 1


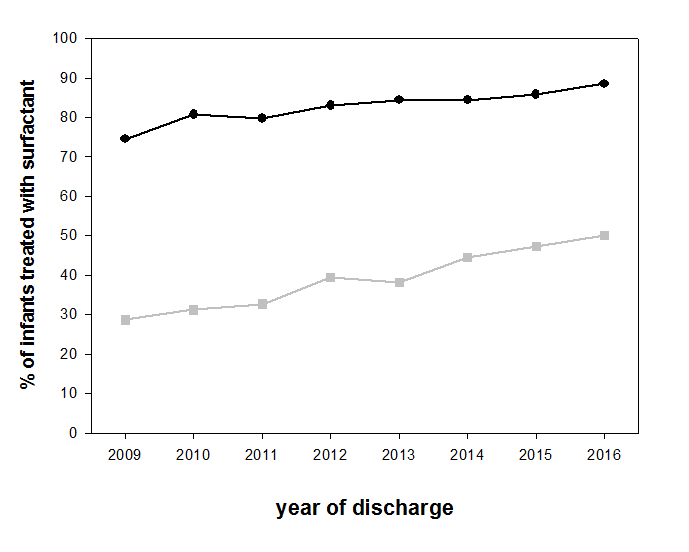


**Supplemental figure 1 legend: Use of LISA strategy during the observational period**

The figure depicts the percentage of infants receiving surfactant treatment (black line) and the proportion of surfactant treated infants who had the LISA procedure (grey line) during the observational period 2009-2016. Total numbers of included infants per year of discharge were 2009: 337, 2010: 650, 2011: 747, 2012: 1026, 2013: 1021, 2014: 1222, 2015: 1399, 2016: 1130.
